# Modelling health belief predictors of oral health and dental anxiety among adolescents based on the Health Belief Model: a cross-sectional study

**DOI:** 10.1186/s12889-020-09784-1

**Published:** 2020-11-23

**Authors:** Bilu Xiang, Hai Ming Wong, Antonio P. Perfecto, Colman P. J. McGrath

**Affiliations:** 1 Department of Paediatric Dentistry, Faculty of Dentistry, The University of Hong Kong, 2/F The Prince Philip Dental Hospital, 34 Hospital Road, Sai Ying Pun, Hong Kong, SAR China; 2 Department of Dental Public Health, Faculty of Dentistry, The University of Hong Kong, Hong Kong, SAR China

**Keywords:** Dental anxiety, Health belief model, Path analysis, Oral health behavior, Oral health, Adolescent

## Abstract

**Background:**

A vicious cycle exists between dental anxiety, oral health behaviors and oral health status. Based on previous research, psychological factors of the Health Belief Model (HBM) are associated with oral health behaviors and oral health, and are likely involved in this cycle. However, little is known about the relationship between HBM factors and dental anxiety of adolescents. The purpose of this cross-sectional study was to investigate the relationship between health belief factors, oral health and dental anxiety based on the constructs of the HBM.

**Methods:**

1207 Grade 2 students from 12 secondary schools in Hong Kong were randomly selected and measured for the decayed, missing and filled permanent teeth (DMFT) index. Data for oral health behaviors, HBM constructs and dental anxiety were collected using questionnaires. The hierarchical entry of explanatory variables into logistic regression models estimating prevalence odds ratios (POR) were analyzed and 95% confidence intervals (95% CI) for DMFT and dental anxiety were generated. Path analysis was used to evaluate the appropriateness of the HBM as predictors for oral health behaviors, DMFT and dental anxiety.

**Results:**

Based on the full model analysis, individuals with higher perceived susceptibility of oral diseases (POR: 1.33, 95% CI: 1.14–1.56) or girls or whose mother received higher education level were likelier to have a DMFT≥1, while those with higher perceived severity (POR: 1.31, 95%CI: 1.09–1.57), flossing weekly, DMFT≥1 or higher general anxiety level statistically increases the possibility of dental anxiety. The results from path analysis indicated that stronger perceived susceptibility, greater severity of oral diseases, less performing of oral health behaviors and a higher score of DMFT were directly related to increased dental anxiety level. Other HBM variables, such as perceived susceptibility, self-efficacy beliefs, cues to action and perceived barriers, might influence dental anxiety through oral health behaviors and caries status.

**Conclusions:**

Clarifying the propositional structures of the HBM may help the future design of theory-based interventions in reducing dental anxiety and preventing dental caries.

**Supplementary Information:**

**Supplementary information** accompanies this paper at 10.1186/s12889-020-09784-1.

## Background

A vicious cycle of dental anxiety, oral health behavior and oral health status has been hypothesized [1]. The vicious cycle theory is proposed that dental anxiety plays a role in the dental avoidance pattern, which in turn, leads to untreated diseases, causing the deterioration of dental anxiety [2]. Nevertheless, findings from another study do not support this downward spiral [3]. The factors which play the major roles and/or initiate the cycle remain unclear. A 3-year cohort study has demonstrated the role of Decayed Missing Filled Teeth (DMFT) scores in the development of dental anxiety, which brings an idea to the mechanism of the vicious cycle [4]. Additionally, multifaceted socio-economical and psychosocial aspects are involved in the onset of dental anxiety [1]. Psychological factors such as personality traits or attachment patterns are also important in the development and persistence of dental anxiety [5, 6]. Children with low psychological functioning tend to have higher levels of dental anxiety and increased social problems [5]. Moreover, self-rated oral health status can trigger dental anxiety which is mediated by certain cognitive vulnerabilities, such as threat or disgust [7]. Signs of depression and anxiety in adolescents [6], as well as higher psychological distress [8], are highly correlated to dental anxiety.

Dental anxiety among youth is a common problem in dental practice. The prevalence of dental anxiety among adolescents ranges from 9.4 to 19% [9]. Almost half of the adolescents in Hong Kong have experience of caries (DMFT≥1) [10, 11], which is similar to some European countries and the United States [12, 13]. Adolescence is a transitional phase from childhood to adulthood, with biological and psychological developmental changes occurring, such as social-networking [14]. In a retrospective study, 22% of respondents reported that their dental anxiety emerged in adolescence [15]. In establishing their health-related behavior and attitudes, dental avoidance in adolescents has the potential to influence their oral health in the short-term and long-term [16].

Psychosocial factors, such as “intention”, “social influences”, and “self-efficacy”, have been identified as important modifiable determinants of tooth brushing frequencies of adolescents [17]. The Health Belief Model (HBM) is one of the psychological theories, which posits that one engages in particular health behaviors based on his belief towards susceptibility to illness and severity, and the perception that there are more benefits over barriers to taking action against illness [18, 19]. Previous research has found that the HBM can predict tooth brushing, flossing and dental visit behaviors [20, 21]. To reinforce the predictability of the HBM, self-efficacy was added into the model to extend the original HBM and was further demonstrated as the strongest predictor of health behaviors in this model [22, 23]. Studies have demonstrated that stronger self-efficacy beliefs and greater perceived severity of oral diseases were related to increased tooth brushing frequency, which in turn was associated with better oral health status [24]. The HBM has not adequately considered the role of emotionality in performing health behaviors, since negative emotions, such as fear or sad moods, might be related to perceived susceptibility and perceived severity [23, 24]. Regarding breast cancer research, individuals with high anxiety levels were found associated with higher perceived severity scores and lower self-efficacy scores [25]. The HBM has also been applied in mental health and anxiety relief contexts [26]. Nevertheless, we are unaware of studies investigating the importance of HBM variables in oral health and dental anxiety contexts.

The objectives of the study were (a) to identify psychological factors contributing to oral health and dental anxiety based on the HBM and (b) to explore the direct and indirect associations of the HBM factors on oral health and dental anxiety via oral health behaviors among Hong Kong adolescents. To the best of our knowledge, this is the first study employing a theoretical model to explore HBM constructs involved in dental anxiety via oral health behaviors and oral health status. A well-known conceptual model of influences on health-related behaviors has been described by Janz and Becker et al. [27]. Based on the previous model, we hypothesized that oral health beliefs (as conceptualized by HBM) involving higher susceptibility, greater severity, more barriers, fewer perceived benefits and weaker self-efficacy, would be associated with increased dental anxiety scores directly or indirectly through oral health behaviors and oral health status.

## Methods

### Participants and sampling

The study was approved by the Institutional Review Board of the University of Hong Kong/Hospital Authority Hong Kong West Cluster (HKU/HA HKW IRB) (IRB HKU: UW18–029). We hypothesized the prevalence of dental anxiety in the adolescent population as 19.5% based on previous studies [9]. The percentage frequency of the estimated dental anxiety was set at 19.5% with confidence limits of ±2.5% and a significant level set at 5%. The sample size was calculated for 965 subjects. Accounting for an 85% response rate, 1136 subjects were required for recruitment. A list of government-funded secondary schools was retrieved from the official website of the Education Bureau, Hong Kong Special Administrative Region (http://www.edb.gov.hk). All secondary schools were coded respectively in the list of their district area (there were four districts of the Hong Kong SAR, i.e. New Territories West, New Territories East, Kowloon and Hong Kong Island). Three schools were randomly selected from each of the four districts using the bowl method, given that there were approximately 100 Grade 2 students in each secondary school. The inclusion criterion included every Grade 2 student from the 12 invited schools. Students with severe systemic diseases, physical, or psychological disabilities were excluded. All eligible adolescents in the participating schools were approached. Written informed consents from parents were obtained prior to their child’s participation. The data were collected through self-reported questionnaires and clinical oral examinations from September 2018 to November 2018.

### Measures

The questionnaire was filled by participants under the supervision of the teacher-in-charge in order to prevent student interaction and maintain data integrity. Age and the gender of participants were requested. The following oral health-related behaviors were measured: frequency of tooth brushing (1. Less than twice a day; 2. Twice or more a day), flossing frequency (1. Never or less than once a week; 2. Once or more a week), sugar consumption (1. Several times a week or daily; 2. Rare) and dental visits (1. No regular dental visit; 2. Have an annual dental visit). Each beneficial behavior scored 1 while discouraged behavior scored 0. The oral health behavior (OHB) score was calculated by summing up the scores of the four beneficial behaviors (ranged from 0 to 4), with a higher score indicating a higher level of oral health behavior.

The constructs of the HBM were measured using a validated questionnaire, Oral Health Behavior Questionnaire for Adolescents based on the Health Belief Model (OHBQAHBM), which consists of 35 items related to 6 interrelated components of the HBM; Perceived Susceptibility (2 items), Perceived Benefits (7 items), Perceived Barriers (6 items), Cues to Action (3 items), Perceived Severity (7 items) and Self-efficacy (10 items) [28]. Each item was scored on a scale from 1 to 5 points and the average score for each subscale was calculated thereby representing the individual’s belief towards that specific component. For each subscale, a higher average score indicates a stronger feeling towards its corresponding component.

Dental anxiety was assessed using a validated questionnaire, the Modified Child Dental Anxiety Scale consisting of 8 questions [29]. Responses were scored from 1 to 5 points, giving a total score of 8–40. A higher score indicates a higher dental anxiety level. The total score was categorized into no anxiety (8–19), mild to moderate anxiety (20–25) and severe anxiety (26–40) for analysis [30]. General anxiety levels were measured using the Chinese version of the Generalized Anxiety Disorder-7 [31]. In the 7-item self-rating questionnaire, each item is scored 0–4 points, giving a total range from 0 to 28. A higher score indicates a higher general anxiety level.

Two trained and calibrated dentists conducted dental examinations in schools using dental mirrors with added lights and Community Periodontal Index probes. Dental caries diagnosis was determined according to the criteria of WHO [32]. DMFT (number of decayed, missing, and filled teeth due to caries) score was calculated. To avoid measurement bias, the clinical examinations were performed unannounced in advance. 10% of children from each school were randomly selected and re-examined on the same day. Acceptable intra- and inter-examiner reliability was achieved (kappa = 0.90–0.94).

### Data analysis

The percentage of missing values of the questionnaire was 0.3–7.0%. For eligible participants, an MCAR (missing completely at random) analysis in SPSS was undertaken to test whether data were missing at random. The *p*-value for the MCAR analysis were all > 0.05, signifying that our data were missing completely at random. The expectation maximization algorithm was used to replace the missing values with predicted values.

**Fig. 1:**
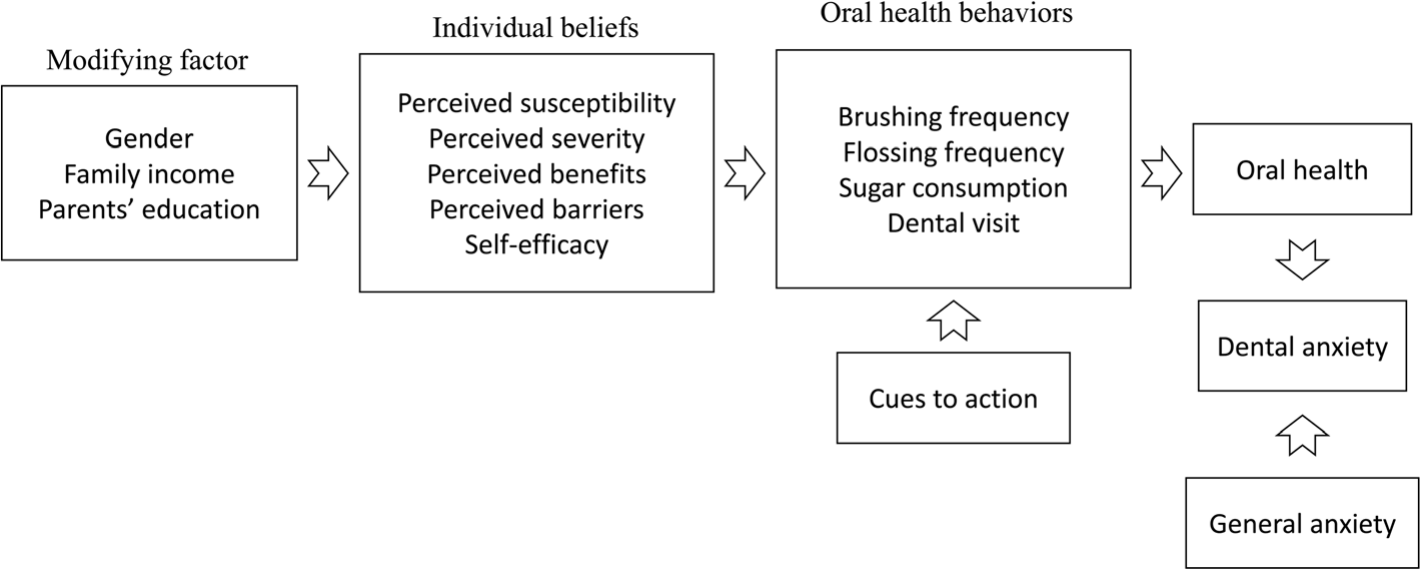
Theoretical model for the study of the health belief model to predict oral health status and dental anxiety (Adapted from Janz & Becker, 1984 [17])

Correlation tests confirmed weak associations among the HBM factors, oral health and dental anxiety (Spearman’s Rho correlation range 0.1–0.4). Variables were not excluded due to collinearity. The comparison of DMFT and dental anxiety between different groups was assessed using chi-square test. The column proportion test was performed to identify whether the prevalence of DMFT and severe dental anxiety in that column was significantly different from other columns. Unadjusted associations between independent variables related to DMFT/ severe dental anxiety were estimated through the odds ratio (OR) and 95% confidence interval (CI). A normality distribution test for general anxiety score, DMFT and HBM variables was used. Since the data were not normally distributed, a Mann-Whitney U test was used to compare the median between groups. Blocks of explanatory variables were entered into a binary logistic regression model using a hierarchical methodology, as predicated by our conceptual model (Fig. 1). The dependent variable of these models were DMFT ≥1 or DMFT = 0 and the existence of severe dental anxiety. The HBM construct factors were entered into Model 1. The modifying factors were entered into Model 2 and oral health behaviors entered into Model 3. For DMFT, the severe dental anxiety level was entered into Model 4. The full model of DMFT (Model 5) comprised the factors in Model 1–4. For severe dental anxiety, DMFT was entered into Model 4 and general anxiety entered into Model 5. The full model of dental anxiety (Model 6) comprised all factors. It is important to note that the full model was built based on a priori selection of covariates according to the conceptual model (Fig. 1) as opposed to covariate selection based upon bivariate statistics. The degree of attenuation was calculated by the 1–[ln (adjusted OR)/ln (unadjusted OR)] formula [33]. In the regression analysis, the HBM factors were continuous variables, whereas gender, parents’ education level, family income and oral health behaviors were categorical variables. The model fit of the regression model was assessed using the Hosmer-Lemeshow test [34]. A *p*-value of the Hosmer-Lemeshow test larger than 0.05 indicated a good fit. The above mentioned statistical analysis was conducted using SPSS 25.0. The chosen level of significance of all the statistical tests was *p* <  0.05 (two-tailed).

To explore the relationship between HBM variables, general anxiety, OHB and DMFT, a path analysis was performed using AMOS 22.0. All variables in the path analysis model were included as continuous variables. The univariate distributions of all variables in the theoretical path model were checked for normality and the skewness and kurtosis values were also measured. Because of the presence of non-normally distributed variables, the path model was evaluated using the Bollen-Stine bootstrapping procedure [35]. In this model, oral hygiene beliefs were posited to be related to dental anxiety both directly and indirectly through oral health behaviors and oral health status. If the *p*-value of the chi-square statistics (χ^2^) exceeded 0.05, the hypothesized path analysis was retained. The model fit was evaluated using multiple fit indices, such as the comparative fit index (CFI), goodness-of-fit index (GFI), Tucker-Lewis index (TLI), the root mean square error of approximation (RMSEA) and the standardized root mean squared residual (SRMR). Cut-offs to consider the model a good fit to the data were CFI > 0.90, TLI > 0.90, RMSEA < 0.06 and SRMR< 0.08 [36].

## Results

### Sample characteristics

Of the 1207 eligible participants, 1159 participated in clinical examinations and returned questionnaires (response rate = 96%). The mean age of the participants was 14.32 ± 0.68 and the proportion of girls were 46.6%. The prevalence of severe dental anxiety [30] among Hong Kong adolescents was 13.7%. Nearly half of adolescents (45.0%) had a DMFT ≥1 (Table 1). 67.9% of adolescents brushed their teeth at least twice a day, but only 20.3% flossed weekly. Most adolescents (81.7%) consumed sugar every week and less than a quarter (23.3%) had annual dental visitation plans. A high proportion of adolescents with DMFT ≥1 were girls with highly educated parents who had stronger perceived susceptibility of oral diseases and more perceived barriers towards performing OHB (Table 1). Dental anxiety among participants was associated with being a girl, lower flossing rates, higher sugar consumption rates, DMFT ≥1, stronger perceived susceptibility, stronger perceived severity, lower self-efficacy and higher general anxiety levels (Table 1).

**Table 1:** The relationship between dental anxiety and oral health behaviors, oral health status, HBM variables and general anxiety

Variable	Total group	DMFT = 0	DMFT≥1	OR (95% CI)	No or mild dental anxiety	Severe dental anxiety	OR (95% CI)
Gender %							
Boys	619 (53.4)	369 (57.9)	250 (47.9)*	1	546 (54.7)	73 (45.9)*	1
Girls	540 (46.6)	268 (42.1)	272 (52.1)*	1.50 (1.19–1.89)	453 (45.3)	86 (54.1)*	1.42 (1.02–1.99)
Father’s education level %							
Elementary school	84 (7.8)	39 (6.6)	45 (9.3)	1	68 (7.3)	16 (10.5)	1
High school	741 (68.7)	400 (67.3)	341 (70.3)	0.74 (0.47–1.16)	634 (68.5)	107 (69.9)	0.72 (0.40–1.28)
College or above	254 (23.5)	155 (26.1)	99 (20.4)*	0.55 (0.34–0.91)	224 (24.2)	30 (19.6)	0.57 (0.29–1.11)
Mother’s education level %							
Elementary school	128 (11.7)	54 (8.9)	74 (15.0)*	1	108 (11.5)	20 (12.9)	1
High school	740 (67.5)	410 (67.9)	330 (66.9)	0.59 (0.40–0.86)	633 (67.2)	107 (69.0)	0.91 (0.54–1.53)
College or above	229 (20.9)	140 (23.2)	89 (18.1)*	0.46 (0.30–0.72)	201 (21.3)	28 (18.1)	0.75 (0.41–1.40)
Monthly family income %							
HK$15,000 or below	183 (18.1)	91 (16.3)	92 (20.3)	1	147 (16.9)	35 (24.6)*	1
HK$15,001 -50,000	688 (67.9)	383 (68.5)	305 (67.2)	0.79 (0.57–1.09)	595 (68.4)	93 (65.5)	0.66 (0.43–1.01)
HK$50,001 or above	142 (14.0)	85 (15.2)	57 (12.6)	0.66 (0.43–1.03)	128 (14.7)	14 (9.9)	0.46 (0.24–0.89)
Tooth brushing behaviors %							
Once a day or less often	372 (32.1)	201 (31.6)	171 (32.8)	1	310 (31.0)	61 (38.4)	1
Twice or more a day	787 (67.9)	436 (68.4)	351 (67.2)	0.95 (0.74–1.21)	689 (69.0)	98 (61.6)	0.72 (0.51–1.02)
Flossing behavior %							
Never or less than once a week	924 (79.7)	503 (79.0)	421 (80.7)	1	790 (79.2)	132 (83.0)	1
At least once a week	235 (20.3)	134 (21.0)	101 (19.3)	0.47 (0.67–1.20)	208 (20.8)	27 (17.0)	0.78 (0.50–1.21)
Sugar consumption %							
Rare or less than once a week	212 (18.3)	509 (79.9)	438 (83.9)	1	191 (19.1)	20 (12.6)*	1
Several times a week or daily	947 (81.7)	128 (20.1)	84 (16.1)	1.30 (0.97–1.78)	807 (80.9)	139 (87.4)*	1.64 (1.00–2.70)
Annual dental visit %							
No	889 (76.7)	478 (75.0)	411 (78.7)	1	724 (74.9)	127 (82.5)*	1
Yes	270 (23.3)	159 (25.0)	111 (21.3)	0.81 (0.62–1.08)	243 (25.1)	27 (17.5)*	0.63 (0.41–0.98)
Variable	Total group	DMFT = 0	DMFT ≥1	p	No or mild dental anxiety	Severe dental anxiety	*p*
Perceived susceptibility (Mean ± SD)^a^	2.7 ± 0.9	2.5 ± 0.9	2.8 ± 0.9	< 0.001	2.6 ± 0.9	2.9 ± 0.9	< 0.01
Perceived severity (Mean ± SD)^a^	3.7 ± 0.9	3.7 ± 0.9	3.7 ± 0.9	0.24	3.7 ± 0.9	3.8 ± 0.8	0.03
Perceived benefits (Mean ± SD)^a^	3.7 ± 0.6	3.7 ± 0.6	3.7 ± 0.6	0.54	3.7 ± 0.6	3.7 ± 0.6	0.65
Perceived barriers (Mean ± SD)^a^	2.3 ± 0.8	2.2 ± 0.8	2.4 ± 0.8	0.001	2.2 ± 0.8	2.4 ± 0.8	0.02
Cues to action (Mean ± SD)^a^	2.1 ± 0.9	2.1 ± 0.9	2.1 ± 0.9	0.90	2.1 ± 0.9	2.0 ± 0.9	0.22
Self-efficacy (Mean ± SD)^a^	3.5 ± 1.0	3.5 ± 1.0	3.4 ± 1.0	0.27	3.5 ± 1.0	3.3 ± 1.0	< 0.05
General anxiety score (Mean ± SD)^a^	4.8 ± 5.2	–	–	–	4.4 ± 5.0	7.4 ± 5.5	< 0.001
Variable	Total group	DMFT = 0	DMFT ≥1	OR (95% CI)	No dental anxiety	Dental anxiety	OR (95% CI)
Oral health (DMFT) %							
DMFT = 0	637 (55.0)	–	–	–	569 (57.0)	67 (42.1)*	1
DMFT ≥1	522 (45.0)	–	–	–	439 (43.0)	92 (57.9)*	1.82 (1.30–2.56)

*Note. P-value < 0.05 (column proportion test)

^a^Note. Mann-Whitney U test was used given non-normal distribution

The Hosmer-Lemeshow test showed a good model fit of all the logistic regression analysis. For an unadjusted model of HBM variables, every increase of one unit in perceived susceptibility resulted in 1.44 times the odds for DMFT ≥1 (Table 2, Model 1). The addition of modifying factors to HBM variables attenuated the effect of perceived susceptibility on DMFT by 14% (Table 2, Model 2), while the addition of oral health behavior variables to HBM variables attenuated the odds by 8% (Table 2, Model 3). The OR was attenuated by 3% with the addition of severe dental anxiety (Table 2, Model 4). A strong perceived susceptibility persisted as a risk indicator for DMFT ≥1 in the final model, which included all covariates. In the full model, the odds of perceived susceptibility was attenuated by 25% (Table 2, Model 5). In addition, girls, low education level of mothers and having severe dental anxiety were also significantly associated with DMFT ≥1 in the full model (Table 2, Model 5).

**Table 2:** Multivariable models evaluating risk indicators for DMFT ≥1 among adolescents

	Model 1 (POR, 95% CI)	Model 2 (POR, 95% CI)	Model 3 (POR, 95% CI)	Model 4	Model 5 (POR, 95% CI)
Perceived susceptibility	1.44 (1.25–1.65)*	1.36 (1.16–1.59)*	1.39 (1.21–1.61)*	1.42 (1.24–1.63)*	1.31 (1.12–1.54)*
Perceived severity	1.00 (0.87–1.15)	0.94 (0.80–1.11)	1.01 (0.87–1.16)	0.98 (0.85–1.14)	0.92 (0.78–1.09)
Perceived benefits	0.98 (0.79–1.20)	1.05 (0.83–1.33)	0.97 (0.79–1.20)	0.98 (0.79–1.20)	1.05 (0.83–1.33)
Perceived barriers	1.16 (0.97–1.39)	1.16 (0.95–1.42)	1.15 (0.95–1.39)	1.13 (0.95–1.36)	1.16 (0.93–1.44)
Cues to action	0.96 (0.84–1.10)	1.00 (0.86–1.17)	0.98 (0.85–1.13)	0.97 (0.85–1.12)	1.02 (0.87–1.19)
Self-efficacy	1.02 (0.89–1.15)	1.00 (0.87–1.16)	1.01 (0.88–1.16)	1.02 (0.90–1.16)	1.00 (0.86–1.17)
Sex					
Boy	–	1	–		1
Girl	–	1.63 (1.25–2.12)*	–		1.64 (1.24–2.17)*
Father’s education level					
Elementary school	–	1	–		1
High school	–	0.76 (0.46–1.25)	–		0.73 (0.44–1.21)
College or above	–	0.66 (0.37–1.21)	–		0.64 (0.35–1.17)
Mother’s education level					
Elementary school	–	1	–		1
High school	–	0.58 (0.38–0.89)*	–		0.58 (0.38–0.91)*
College or above	–	0.56 (0.32–0.98)*	–		0.54 (0.31–0.96)*
Family income per month					
HK$15,000 or below	–	1	–		1
HK$15,001 -50,000	–	1.00 (0.69–1.43)	–		1.02 (0.71–1.49)
HK$50,001 or above	–	1.01 (0.61–1.68)	–		1.07 (0.64–1.79)
Tooth brushing behavior					
Once a day or less often	–	–	1		1
Twice or more a day	–	–	0.96 (0.73–1.28)		0.94 (0.68–1.30)
Flossing behavior					
Never or less than once a week	–	–	1		1
At least once a week	–	–	0.99 (0.73–1.35)		1.01 (0.71–1.42)
Sugar consumption					
Rare or less than once a week	–	–	1		1
Several times a week or daily	–	–	1.23 (0.69–1.26)		1.09 (0.75–1.57)
Annual dental visit					
No	–	–	1		1.08 (0.76–1.53)
Yes	–	–	0.93 (0.69–1.26)		
Dental anxiety level					
No or mild				1	1
Severe				1.66 (1.17–2.35)*	1.51 (1.03–2.21)*
−2 Log likelihood	1558	1261	1508	1549	1218
Nagelkerke R^2^	0.042	0.072	0.042	0.051	0.080

Note: *p < 0.05

In the unadjusted model, the increase in perceived susceptibility, perceived severity, perceived barriers and significantly resulted in a higher chance of severe dental anxiety (Table 3, Model 1). In the full model, only perceived susceptibility and perceived severity remained significantly associated with severe dental anxiety. The odds of perceived susceptibility and perceived severity on severe dental anxiety were 1.27 and 1.38, which were attenuated by 11% and intensified by 10% after adjusting for confounding factors, respectively (Table 3, Model 6). In addition, tooth brushing behavior, DMFT and general anxiety remained statistically associated with severe dental anxiety in the full model (Table 3, Model 6).

**Table 3:** Multivariable models evaluating risk indicators for severe dental anxiety among adolescents

	Model 1 (POR, 95% CI)	Model 2 (POR, 95% CI)	Model 3 (POR, 95% CI)	Model 4 (POR, 95% CI)	Model 5 (POR, 95% CI)	Model 6 (POR, 95% CI)
Perceived susceptibility	1.31 (1.07–1.59)*	1.38 (1.11–1.72)*	1.27 (1.04–1.56)*	1.25 (1.02–1.53)*	1.26 (1.03–1.54)*	1.27 (1.01–1.60)*
Perceived severity	1.34 (1.08–1.66)*	1.40 (1.09–1.78)*	1.39 (1.11–1.73)*	1.35 (1.08–1.68)*	1.25 (1.00–1.56)*	1.38 (1.07–1.78)*
Perceived benefits	1.02 (0.76–1.37)	1.00 (0.73–1.38)	0.97 (0.72–1.32)	1.02 (0.75–1.37)	1.01 (0.74–1.37)	0.93 (0.66–1.30)
Perceived barriers	1.36 (1.06–1.73)*	1.29 (0.98–1.70)	1.30 (0.99–1.70)	1.34 (1.04–1.71)*	1.33 (1.04–1.71)*	1.17 (0.86–1.59)
Cues to action	0.85 (0.70–1.05)	0.92 (0.74–1.15)	0.89 (0.72–1.10)	0.86 (0.70–1.05)	0.88 (0.72–1.09)	0.97 (0.77–1.22)
Self-efficacy	0.89 (0.74–1.06)	0.88 (0.72–1.08)	0.95 (0.77–1.16)	0.89 (0.74–1.06)	0.95 (0.79–1.14)	1.06 (0.84–1.33)
Sex						
Boy	–	1	–	–	–	1
Girl	–	1.30 (0.89–1.89)	–	–	–	1.14 (0.76–1.70)
Father’s education level						
Elementary school	–	1	–	–	–	1
High school	–	0.79 (0.42–1.50)	–	–	–	0.87 (0.45–1.68)
College or above	–	0.76 (0.34–1.69)	–	–	–	0.80 (0.35–1.81)
Mother’s education level						
Elementary school	–	1	–	–	–	1
High school	–	1.36 (0.75–2.48)	–	–	–	1.50 (0.80–2.80)
College or above	–	1.30 (0.58–2.87)	–	–	–	1.75 (0.77–4.01)
Family income per month						
HK$15,000 or below	–	1	–	–	–	1
HK$15,001 -50,000	–	1.09 (0.76–1.57)	–	–	–	0.76 (0.47–1.22)
HK$50,001 or above	–	1.02 (0.62–1.70)	–	–	–	0.51 (0.24–1.09)
Tooth brushing behavior						
Once a day or less often	–	–	1	–	–	1
Twice or more a day	–	–	0.76 (0.51–1.13)	–	–	0.63 (0.40–0.99)*
Flossing behavior						
Never or less than once a week	–	–	1	–	–	1
At least once a week	–	–	0.97 (0.62–1.54)	–	–	0.97 (0.58–1.61)
Sugar consumption						
Rare or less than once a week	–	–	1	–	–	1
Several times a week or daily	–	–	1.64 (0.98–2.75)	–	–	1.53 (0.86–2.74)
Annual dental visit						
No	–	–	1	–	–	1
Yes	–	–	0.77 (0.48–1.23)	–	–	0.76 (0.44–1.29)
Oral health						
DMFT = 0	–	–	–	1	–	1
DMFT≥1	–	–	–	1.66 (1.17–2.35)*	–	1.58 (1.07–2.35)*
General anxiety	–	–	–	–	1.09 (1.06–1.12)*	1.07 (1.04–1.11)*
−2 Log likelihood	899	757	864	890	1441	1151
Nagelkerke R^2^	0.043	0.065	0.053	0.056	0.090	0.113

Note:*p < 0.05

### Path analysis modeling

**Fig. 2:**
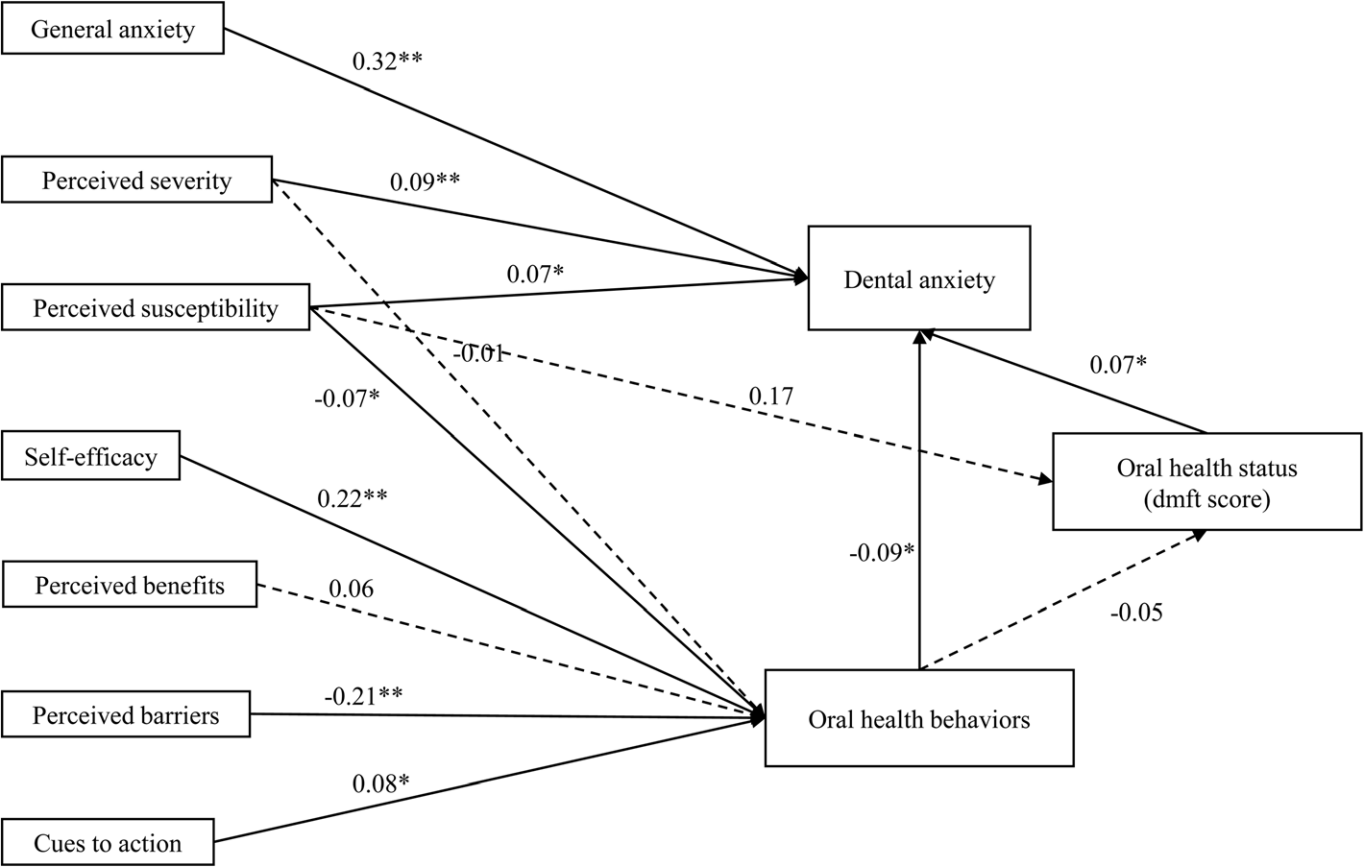
Path analysis of psychological factors as predictors for dental anxiety. Standardized direct path coefficients are presented. Note. Significant differences indicated by ∗∗*p* < 0.01; ∗*p* < 0.05

The model was firstly based on the conceptual model and secondly modified according to the regression results. Three paths were added to the model: one path between perceived susceptibility and dental anxiety; and one path between perceived severity and dental anxiety; furthermore, one path linked the perceived susceptibility to oral health (DMFT). The final model is depicted in Fig. 2 and Table 4. The model was well fitted (TLI = 0.99; CFI = 1.00; RMSEA = 0.01; SRMR = 0.01; Bollen-Stine bootstrap *p* = 0.35). Regarding the direct effect, a significant path was noted from general anxiety to dental anxiety (β = 0.44, *p* <  0.01). Consistent with the regression results, higher perceived susceptibility (β = 0.56, *p* = 0.03) and greater perceived severity (β = 0.72, p <  0.01) were associated with greater dental anxiety. Significant direct paths were also found to OHB from perceived susceptibility (β = − 0.07, *p* = 0.04), self-efficacy (β = 0.20, p <  0.01), perceived barriers (β = − 0.25, p <  0.01) and cues to action (β = 0.08, *p* = 0.01). Regarding the direct effects of OHB and DMFT on dental anxiety, both were significant (β = − 0.74, p <  0.01; β = 0.28, *p* = 0.02).

**Table 4:** Standardized and unstandardized path coefficients of the path analysis model

Effects	Standardized path coefficient (β)	Unstandardized path coefficient	SE	95% CI	Bootstrapping p	R^2^
Oral health behaviors						
Perceived susceptibility	−0.07	−0.07	0.03	−0.12 to −0.00	0.04	0.14
Perceived severity	−0.01	−0.01	0.03	− 0.08 to 0.05	0.72	
Perceived benefits	0.06	0.09	0.05	−0.01 to 0.17	0.08	
Perceived barriers	−0.21	−0.25	0.04	−0.33 to − 0.17	< 0.01	
Cues to action	0.08	0.08	0.03	0.02 to 0.13	0.01	
Self-efficacy	0.23	0.20	0.03	0.15 to 0.25	< 0.01	
Oral health (DMFT)						
Oral health behaviors	−0.05	−0.08	0.06	−0.20 to 0.04	0.09	0.04
Perceived susceptibility	0.17	0.30	0.18	−0.06 to 0.66	0.16	
Dental anxiety						
Perceived susceptibility	0.07	0.56	0.24	0.05 to 1.03	0.03	0.14
Perceived severity	0.09	0.72	0.23	0.31 to 1.18	< 0.01	
General anxiety	0.32	0.44	0.04	0.36 to 0.52	< 0.01	
Oral health (DMFT)	0.07	0.28	0.13	0.04 to 0.54	0.02	
Oral health behaviors	−0.09	−0.74	0.23	−1.18 to −0.30	< 0.01	

Note: 95% CI, 95% confidence interval of the unstandardized path coefficient; SE, standard error of the unstandardized path coefficient

For indirect effects exerted through OHB and DMFT, perceived susceptibility (β = 0.14, *p* < 0.01), self-efficacy beliefs (β = − 0.16, p < 0.01), cues to action (β = − 0.06, p < 0.01) and perceived barriers (β = 0.19, p < 0.01) were statistically significant. The dotted line of Fig. 2 denoted the insignificant paths, but for conceptual reasons, it was decided to retain the paths. The final model explained 14% of variances in oral health behaviors and 14% of variances in dental anxiety.

## Discussion

This study suggests that HBM factors are risk indicators for caries and dental anxiety among Hong Kong adolescents. After adjusting for socio-demographic factors and behavior covariates, the association of perceived susceptibility with DMFT score and perceived severity in relation to dental anxiety was maintained.

We believe that this is the first study to examine the complex predictors regarding oral health and dental anxiety after accounting for the impact of HBM variables in a path analysis model of data. Our findings suggested that perceived susceptibility, perceived barriers, self-efficacy and cues to action could predict oral health behaviors. The results were in accordance with other studies that perceived barriers, self-efficacy and cues to action played a role in predicting oral health behaviors [37, 38]. Perceived benefit was rarely identified as a significant predictor. Some research also did not support the predictability of perceived severity to behavior change [37, 39]. The reason why the perceived severity was not identified as a predictor might be the adoption of perceived subjective severity in the present study. Perceived severity contained two distinct concepts: subjective severity and objective severity; objective severity played a more important role in predicting oral health behaviors among young adolescents rather than subjective severity [40]. Besides, our study indicated that self-efficacy and perceived barriers were the strongest predictors of oral health behaviors, which confirmed the results from other studies [37, 41, 42]. It was even claimed in a study that reducing barriers was one of the most useful strategies to encourage oral health behaviors [37].

What’s more, our findings suggested that oral health beliefs (including HBM constructs) were associated with dental anxiety directly or indirectly via OHB and oral health. In recent decades, pressure has been placed on therapeutics to reduce patients’ anxiety in the long term without pharmacological use [43, 44]. Psychological treatments have displayed better improvement in dental anxiety prevention in the long term compared to the use of pharmaceuticals [45]. In our study, we identified the role of HBM psychological constructs on the severity of dental anxiety. Threat-related perceptions based on past experiences may bring negative expectations of dental treatment and trigger dental phobia [46]. From the perspective of the HBM, threat perceptions are based on two beliefs: perceived susceptibility and perceived severity [23]. Perceived susceptibility refers to the chance of obtaining a disease or a painful state; perceived severity refers to one’s belief towards the effect and psychological harm the disease could create [23]. In previous research on preoperative anxiety, perceived severity was a risk factor for increased anxiety levels [47]. Moreover, negative emotions, such as fear and sadness, was found to have a link with the perceived threat [24, 48]. One study proposed a mechanism that the rehearsal of threatening outcomes in the absence of active planning or activation of relevant coping information increased anxious arousal [49]. As proposed by the extended parallel process model, fear, as well as anxiety, were aroused by threat-related emotions, including perceived susceptibility and perceived severity, and those two threat messages played a role in protection intention against diseases [50]. In the present study, perceived severity and perceived susceptibility were positively correlated with dental anxiety directly, whereas perceived susceptibility showed a positive, though nonsignificant direct effect on oral health. These results were consistent with a study on meningitis, in which the perceived susceptibility was the most influential predictor of protective action while perceived severity contributed to a weaker effect [39]. Other variables from the HBM are able to predict dental anxiety via the oral health behavior path. The HBM theory also proposes that if an individual has sufficient self-efficacy, perceived benefits over barriers, and cues to action, he is more likely to perform a behavior [23]. Dental anxiety is a risk factor for caries in children [33] and individuals with poorer oral health practices are correlated with higher dental anxiety levels [51]. Our study results were consistent with previous studies and the HBM variables indicate that they are related to dental anxiety via OHB and caries status.

However, the HBM variables predicted only 14% of the variance in both oral health behaviors and dental anxiety, leaving 86% of the variance unaccounted for. This suggested that HBM factors owned the ability to predict dental anxiety as well as oral health behaviors. But it also indicated that there are other important determinants of healthy behaviors and dental anxiety not yet accounted for by HBM. This points to the need to investigate other determinants that were not accounted for by HBM, such as demographic variables. In addition, most HBM researchers assumed that the individual determinants were only directly related to healthy behaviors and no indirect or mediating effects exist between the variables [23]. Finally, based on the amount of variance that HBM contributed to oral health behaviors and dental anxiety, it would be beneficial to investigate whether the HBM-based strategy is effective in clinical trials in future studies.

### Limitations

One of the major limitations of our study is the cross-sectional study design of the work. Given the nature of the design, a causal relationship between psychological factors and dental anxiety cannot be determined. Thus, future work is necessary to test this relationship using a longitudinal study design. Another limitation of our study is the use of self-reported measures to assess oral health behaviors. There is a possibility that social desirability may introduce bias. The third limitation of our findings is that it may not be generalizable to older adolescents as differences in psychological and physical status exist between early adolescents and late adolescents [52]. Regardless, the importance of the HBM in oral health and disease should be investigated further.

## Conclusions

The present study suggests directions and further steps to be taken to reduce dental anxiety and improve oral health status in adolescents. The need for cognitive-behavioral interventions is further evidenced by the fact that 2/3 of adolescents brushed their teeth as recommended (at least twice a day) but only 20.0% of adolescents flossed weekly. Most adolescents had a high frequency of sugar intakes and did not have plans for annual dental visitation. Moreover, our study found a relatively high prevalence of dental anxiety (40.5%) and DMFT ≥1 (45.0%). A high prevalence of dental anxiety has been shown to result in increased dental avoidance and poorer oral health outcomes. Our analysis of dental anxiety and oral health from a cognitive theory model perspective, such as the HBM, provides a clearer explanation for one of the mechanisms involved in oral health and dental anxiety among adolescents. Thus, there is a tangible application for the implementation of theory-based behavioral interventions targetting the promotion of oral health behaviors in schools as an alternative strategy in reducing dental anxiety and prevent oral diseases in adolescents.

## Supplementary Information

**Additional file 1.** Questionnaire.

## Data Availability

The datasets used and/or analyzed for the current study are available from the corresponding author on reasonable request.

## Abbreviations

HBM: Health Belief Model
DMFT: Missing and filled permanent teeth
POR: Prevalence odds ratios
CI: Confidence interval
OHB: Oral health behavior
CFI: Comparative fit index
GFI: Goodness-of-fit index
TLI: Tucker-Lewis index
RMSEA: The root mean square error of approximation
SRMR: The standardized root mean squared residual
