# Supplementary material for: Modelling health belief predictors of oral health and dental anxiety among adolescents based on the Health Belief Model: a cross-sectional study
Source: BMC Public Health. 2020 Nov 23;20:1755. doi: 10.1186/s12889-020-09784-1 (PMC7686751; doi:10.1186/s12889-020-09784-1)
Supplement: Supplementary file 1 — Additional file 1. Questionnaire. [file 12889_2020_9784_MOESM1_ESM.docx]

**Questionnaire**

**Part I: Basic information**

1. Date: _________________

2. Name: ________________

3. School: _______________

4. Gender: □_1_ Male □_2_ Female

5. Date of birth: _______________(DD/MM/YYYY)

**Part II: Oral health behavior**

1. How often did you brush your teeth in the past month?

□_1_ Less than twice a day □_2_ Twice or more a day

2. How often did you use dental floss in the past month?

□_1_ Never or less than once a week □_2_ Once or more a week

3. How often did you consume sugar like candy, chocolate or biscuits?

□_1_ Several times a week or daily □_2_ Rare

4. Do you have any plan for regular dental check-up?

□_1_ No regular dental visit □_2_ Yes. Have an annual dental visit

**Part III. Cognition related to oral health knowledge (Oral health behavior mentioned below refers to tooth brushing and flossing, please tick √at the appropriate box) (HBM construct)**

| A. | Strongly disagree | Disagree | Neutral | Agree | Strongly agree |
| --- | --- | --- | --- | --- | --- |
| 1. There is a chance that I will get caries. | □_1_ | □_1_ | □_3_ | □_4_ | □_5_ |
| 2. There is a chance that I will have periodontal disease. | □_1_ | □_2_ | □_3_ | □_4_ | □_5_ |
| 3. I think brushing and flossing can make teeth healthier. | □_1_ | □_2_ | □_3_ | □_4_ | □_5_ |
| 4. I think brushing and flossing can prevent oral diseases from happening. | □_1_ | □_2_ | □_3_ | □_4_ | □_5_ |
| 5. I think brushing and flossing can make teeth look good. | □_1_ | □_2_ | □_3_ | □_4_ | □_5_ |
| 6. I think brushing and flossing can keep breath fresh. | □_1_ | □_2_ | □_3_ | □_4_ | □_5_ |
| 7. I think brushing and flossing can prevent inconvenient eating caused by oral diseases. | □_1_ | □_2_ | □_3_ | □_4_ | □_5_ |
| 8. I think brushing and flossing can help me avoid spending more time on dental treatment in the future. | □_1_ | □_2_ | □_3_ | □_4_ | □_5_ |
| 9. I think brushing and flossing can help me avoid spending more money on dental treatment in the future. | □_1_ | □_2_ | □_3_ | □_4_ | □_5_ |
| 10. I think it is difficult for me to brush twice a day. | □_1_ | □_2_ | □_3_ | □_4_ | □_5_ |
| 11. I think it’s a waste of time to brush and floss. | □_1_ | □_2_ | □_3_ | □_4_ | □_5_ |
| 12. I think I do not have enough time to have an annual dental visit. | □_1_ | □_2_ | □_3_ | □_4_ | □_5_ |
| 13. I am afraid of undergoing tooth treatment, so I don’t have dental visits annually. | □_1_ | □_2_ | □_3_ | □_4_ | □_5_ |
| 14. I think we have no money at my home, so I don’t have an annual dental visit. | □_1_ | □_2_ | □_3_ | □_4_ | □_5_ |
| 15. I think the dental clinic is far from my home, so I don’t have an annual dental visit. | □_1_ | □_2_ | □_3_ | □_4_ | □_5_ |
| 16. Parents often reminds me of brushing and flossing | □_1_ | □_2_ | □_3_ | □_4_ | □_5_ |
| 17. Classmates often reminds me of brushing and flossing. | □_1_ | □_2_ | □_3_ | □_4_ | □_5_ |
| 18. Teachers often reminds me of brushing and flossing. | □_1_ | □_2_ | □_3_ | □_4_ | □_5_ |
| B. | Not serious | A little serious | Partially serious | Serious | Very serious |
| 19. If I have caries, for me that is… | □_1_ | □_2_ | □_3_ | □_4_ | □_5_ |
| 20. If I have periodontal disease, for me that is… | □_1_ | □_2_ | □_3_ | □_4_ | □_5_ |
| 21. If my teeth do not look good because of oral diseases, for me that is… | □_1_ | □_2_ | □_3_ | □_4_ | □_5_ |
| 22. If I have bad breath because of oral diseases, for me that is… | □_1_ | □_2_ | □_3_ | □_4_ | □_5_ |
| 23. If I can’t sleep well because of oral diseases, for me that is… | □_1_ | □_2_ | □_3_ | □_4_ | □_5_ |
| 24. If I can’t eat my favorite food because of oral diseases, for me that is… | □_1_ | □_2_ | □_3_ | □_4_ | □_5_ |
| 25. If I get laughed at by classmates because of oral diseases, for me that is… | □_1_ | □_2_ | □_3_ | □_4_ | □_5_ |
| C. How confident you are that you will brush your teeth for 2 min twice daily on the circumstances below? | Not confident | A bit confident | Fairly confident | Quite confident | Very confident |
| 26. When you are under a lot of stress | □_1_ | □_2_ | □_3_ | □_4_ | □_5_ |
| 27. During or after experiencing personal problems | □_1_ | □_2_ | □_3_ | □_4_ | □_5_ |
| 28. When you are feeling tired | □_1_ | □_2_ | □_3_ | □_4_ | □_5_ |
| 29. When you don’t feel like it | □_1_ | □_2_ | □_3_ | □_4_ | □_5_ |
| 30. When you are anxious | □_1_ | □_2_ | □_3_ | □_4_ | □_5_ |
| 31. After experiencing family problems | □_1_ | □_2_ | □_3_ | □_4_ | □_5_ |
| 32. When you have other commitments | □_1_ | □_2_ | □_3_ | □_4_ | □_5_ |
| 33. When you feel you don’t have  the time | □_1_ | □_2_ | □_3_ | □_4_ | □_5_ |
| 34. When you are feeling under pressure from school work | □_2_ | □_2_ | □_3_ | □_4_ | □_5_ |
| 35. When you have too much work to do at home | □_1_ | □_2_ | □_3_ | □_4_ | □_5_ |

**Part IV. Dental Anxiety Scale (Please tick the appropriate box)**

| How do you feel about under the following circumstances? | Not worried | Slightly worried | Fairly worried | Worried a lot | Very worried |
| --- | --- | --- | --- | --- | --- |
| 1. going to the dentist generally | □_1_ | □_2_ | □_3_ | □_4_ | □_5_ |
| 2. having your teeth looked at | □_1_ | □_2_ | □_3_ | □_4_ | □_5_ |
| 3. having your teeth scraped and polished | □_1_ | □_2_ | □_3_ | □_4_ | □_5_ |
| 4. having an injection in the gum | □_1_ | □_2_ | □_3_ | □_4_ | □_5_ |
| 5. having a filling | □_1_ | □_2_ | □_3_ | □_4_ | □_5_ |
| 6. having a tooth taken out | □_1_ | □_2_ | □_3_ | □_4_ | □_5_ |
| 7. being put to sleep to have treatment | □_1_ | □_2_ | □_3_ | □_4_ | □_5_ |
| 8. having a mixture of "gas and air" which will help you to feel comfortable for treatment, but which cannot put you to sleep | □_1_ | □_2_ | □_3_ | □_4_ | □_5_ |

**Part V. Anxiety Scale.**

| Over the last 2 weeks, how often have you been bothered by the following problems? (Please tick the appropriate box) | Not at all | Several days | More than half the days | Nearly every day |
| --- | --- | --- | --- | --- |
| 1. Feeling nervous, anxious or on edge | □_1_ | □_2_ | □_3_ | □_4_ |
| 2. Not being able to stop or control worrying | □_1_ | □_2_ | □_3_ | □_4_ |
| 3. Worrying too much about different things | □_1_ | □_2_ | □_3_ | □_4_ |
| 4. Trouble relaxing | □_1_ | □_2_ | □_3_ | □_4_ |
| 5. Being so restless that it is hard to sit still | □_1_ | □_2_ | □_3_ | □_4_ |
| 6. Becoming easily annoyed or irritable | □_1_ | □_2_ | □_3_ | □_4_ |
| 7. Feeling afraid as if something awful might happen | □_1_ | □_2_ | □_3_ | □_4_ |

**Part VI. Other information (Please tick the appropriate box)**

1. Parent education level:

|  | Father | Mother |
| --- | --- | --- |
| Elementary school | □ | □ |
| High school | □ | □ |
| College or above | □ | □ |

2. Monthly family income:

□ HK$15,000 or below

□ HK$15,001-50,000

□ HK$50,001 or above
